# A Review of Emotion Recognition Using Physiological Signals

**DOI:** 10.3390/s18072074

**Published:** 2018-06-28

**Authors:** Lin Shu, Jinyan Xie, Mingyue Yang, Ziyi Li, Zhenqi Li, Dan Liao, Xiangmin Xu, Xinyi Yang

**Affiliations:** School of Electronic and Information Engineering, South China University of Technology, Guangzhou 510641, China; shul@scut.edu.cn (L.S.); xie.jinyan@mail.scut.edu.cn (J.X.); eemingyueyang@mail.scut.edu.cn (M.Y.); eve.llzzyy@gmail.com (Z.L.); li.zhenqi@mail.scut.edu.cn (Z.L.); danl@scut.edu.cn (D.L.);cindy.yang1001@gmail.com (X.Y.)

**Keywords:** emotion recognition, physiological signals, emotion model, emotion stimulation, features, classifiers

## Abstract

Emotion recognition based on physiological signals has been a hot topic and applied in many areas such as safe driving, health care and social security. In this paper, we present a comprehensive review on physiological signal-based emotion recognition, including emotion models, emotion elicitation methods, the published emotional physiological datasets, features, classifiers, and the whole framework for emotion recognition based on the physiological signals. A summary and comparation among the recent studies has been conducted, which reveals the current existing problems and the future work has been discussed.

## 1. Introduction

Emotions, which affect both human physiological and psychological status, play a very important role in human life. Positive emotions help improve human health and work efficiency, while negative emotions may cause health problems. Long term accumulations of negative emotions are predisposing factors for depression, which might lead to suicide in the worst cases. Compared to the mood which is a conscious state of mind or predominant emotion in a time, the emotion often refers to a mental state that arises spontaneously rather than through conscious effort and is often accompanied by physical and physiological changes that are relevant to the human organs and tissues such as brain, heart, skin, blood flow, muscle, facial expressions, voice, etc. Due to the complexity of mutual interaction of physiology and psychology in emotions, recognizing human emotions precisely and timely is still limited to our knowledge and remains the target of relevant scientific research and industry, although a large number of efforts have been made by researchers in different interdisciplinary fields. 

Emotion recognition has been applied in many areas such as safe driving [1], health care especially mental health monitoring [2], social security [3], and so on. In general, emotion recognition methods could be classified into two major categories. One is using human physical signals such as facial expression [4], speech [5], gesture, posture, etc., which has the advantage of easy collection and have been studied for years. However, the reliability can’t be guaranteed, as it’s relatively easy for people to control the physical signals like facial expression or speech to hide their real emotions especially during social communications. For example, people might smile in a formal social occasion even if he is in a negative emotion state. The other category is using the internal signals—the physiological signals, which include the electroencephalogram (EEG), temperature (T), electrocardiogram (ECG), electromyogram (EMG), galvanic skin response (GSR), respiration (RSP), etc. The nervous system is divided into two parts: the central and the peripheral nervous systems (CNS and PNS). The PNS consists of the autonomic and the somatic nervous systems (ANS and SNS). The ANS is composed of sensory and motor neurons, which operate between the CNS and various internal organs, such as the heart, the lungs, the viscera, and the glands. EEG, ECG, RSP, GSR, and EMG change in a certain way when people face some specific situations. The physiological signals are in response to the CNS and the ANS of human body, in which emotion changes according to Connon’s theory [6]. One of the major benefits of the latter method is that the CNS and the ANS are largely involuntarily activated and therefore cannot be easily controlled. There have been a number of studies in the area of emotion recognition using physiological signals. Attempts have been made to establish a standard and a fixed relationship between emotion changes and physiological signals in terms of various types of signals, features, and classifiers. However, it was found that it was relatively difficult to precisely reflect emotional changes by using a single physiological signal. Therefore, emotion recognition using multiple physiological signals presents its significance in both research and real applications. This paper presents a review on emotion recognition using multiple physiological signals. It is organized as follows: the emotion models are analyzed in Section 2. The features extracted from physiological signals especially the emotional relevant features are analyzed in Section 3. The framework of emotion recognition is presented in Section 4, including preprocessing, feature extraction, feature optimization, feature fusion, classification and model evaluation. In Section 5, several physiological signal databases under certain emotional stimulation are stated. A comprehensive summary and comparison was given in Section 6. In Section 7 some drawbacks in current studies are pointed and a discussion regarding the future work is presented.

## 2. Emotion Models

For emotion recognition, the emotions should be defined and accessed quantitatively. The definition of basic emotions was firstly proposed decades ago. However, the precise definition has never been widely acknowledged by psychologists. Psychologists tend to model emotions in two different ways. One is to divide emotions into discrete categories. The other is to use multiple dimensions to label emotions. For emotion elicitation, subjects are given a series of emotionally-evocative materials to induce a certain emotion. During the past few years, pictures, music and films stimulations are the most common materials. Furthermore, some novel methods called situational stimulation are rising in recent years. Computer games or recollection are used to induce emotion. Among these methods, affective Virtual Reality has attracted more and more attentions. 

### 2.1. Discrete Emotion Models 

Ekman [7] regarded emotions as discrete, measurable and physiology-related. He proposed a number of characteristics towards basic emotions. (1) People are born with emotions which are not learned; (2) People exhibit the same emotions in the same situation; (3) People express these emotions in a similar way; (4) People show similar physiological patterns when expressing the same motions. According to the characteristics, he summarized six basic emotions of happy, sad, anger, fear, surprise, and disgust, and viewed the other emotions as the production of reactions and combinations of these basic emotions.

**Figure 1:**
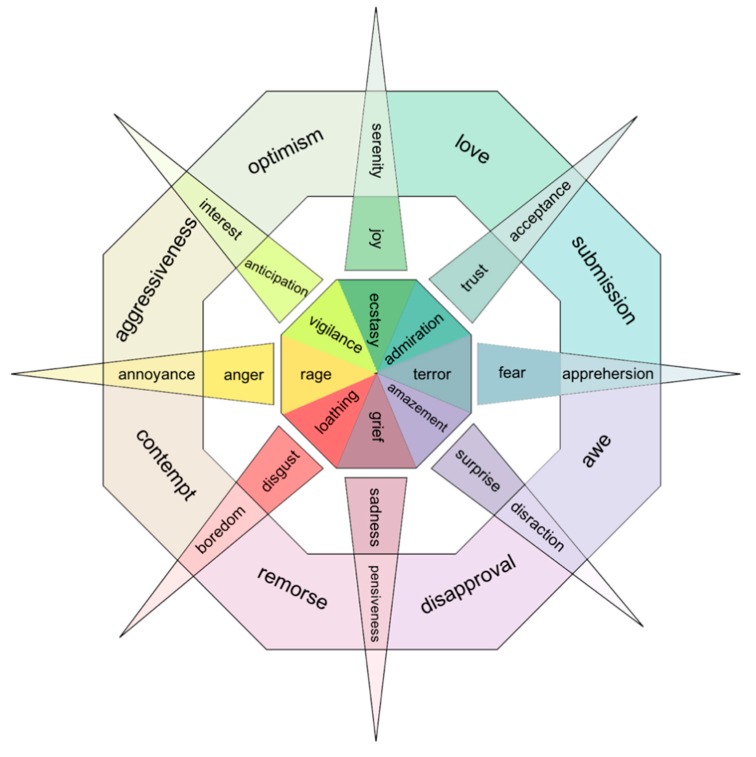
Plutchik’s Wheel of Emotions.

In 1980, Plutchik [8] proposed a wheel model that includes eight basic emotions of joy, trust, fear, surprise, sadness, disgust, anger and anticipation, as shown in Figure 1. This wheel model describes emotions according to intensity, the stronger emotions are in the center while the weaker emotions are at the flower blooms. Just as colors, basic emotions can be mixed to form complex emotions. Izard [9,10] described that: (1) Basic emotions were formed in the course of human evolution; (2) Each basic emotion corresponded to a simple brain circuit and there was no complex cognitive component involved. He then presented ten basic emotions: interest, joy, surprise, sadness, fear, shyness, guilt, angry, disgust, and contempt.

The discrete emotion models have utilized word descriptions for emotions instead of quantitative analysis. It is therefore difficult to analyze complex emotions, such as some mixed emotions that are difficult to be precisely expressed in words and need to be studied quantitatively. 

### 2.2. Multi-Dimensional Emotion Space Model

**Figure 2:**
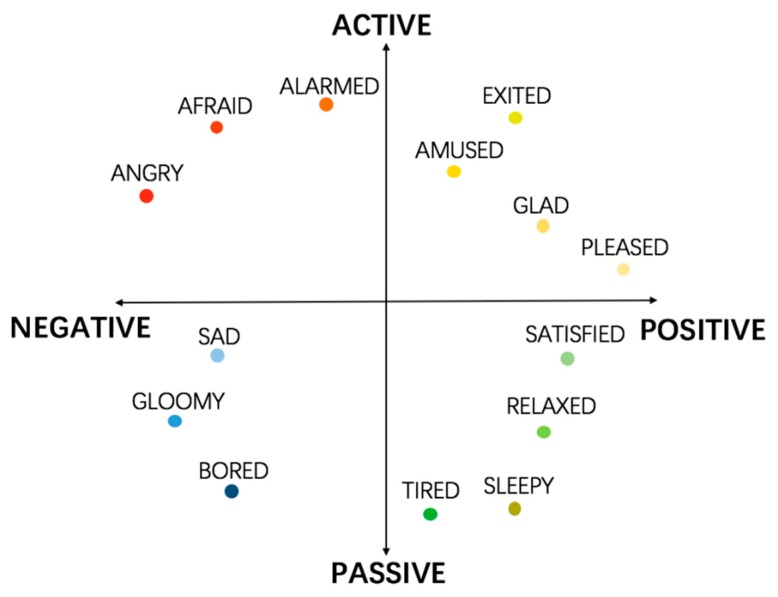
2D emotion space model.

With the deepening of research, psychologists found that there was a certain correlation among separate emotions, such as hatred and hate, pleasure and liking, which represented a certain degree of specific emotional level. On the other hand, the emotions with the same descriptions may have different intensities. For example, happy might be described as a little bit happy or very happy. Therefore, psychologists have tried to construct multi-dimensional emotion space models. Lang [11] investigated that emotions can be categorized in a 2D space by valence and arousal. In his theory, valence ranges from unpleasant (negative) to pleasant (positive), and arousal ranges from passive (low) to active (high), which indicate how strongly human feels. Different emotions can be plotted in the 2D space as shown in Figure 2. For example, anger has negative valence and high arousal while sadness has negative valence and low arousal.

**Figure 3:**
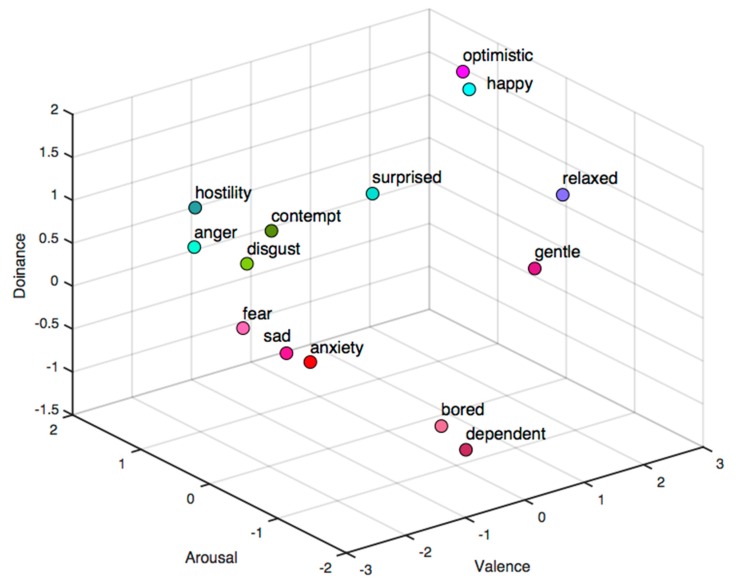
3D emotion space model.

Although it can easily distinguish positive and negative emotions, it will fail in recognizing similar emotions in the 2D emotion space. For example, fear and angry are both inside the zone with negative valence and high arousal. Mehrabian [12] extended the emotion model from 2D to 3D (see Figure 3). The added dimension axis is named dominance ranging from submissive to dominant, which reflects the control ability of the human in a certain emotion. In this dimension, anger and fear can be easily identified as anger is in the dominant axis while fear is in the submissive axis. 

### 2.3. Emotion Stimulation Tools

National Institute of Mental Health [13] proposed the well-known International Affective Picture System (IAPS) in 1997, which provided a series of standardized, emotionally-evocative photographs that can be accessed by everyone. Additionally, in 2005, the Chinese Affective Picture System (CAPS) was proposed [14], which was an important tool for domestic researchers. 

**Figure 4:**
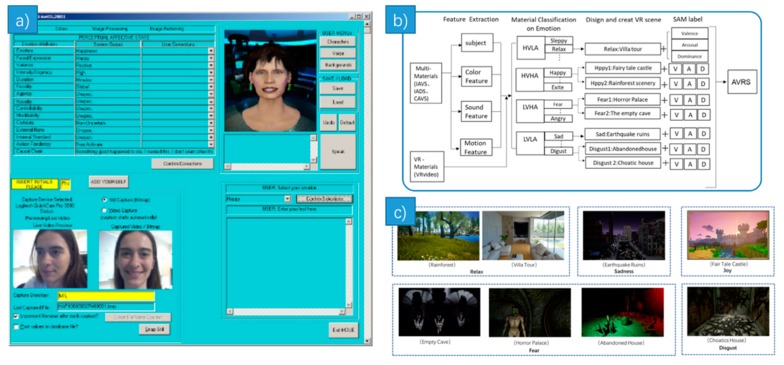
(**a**) MAUI—Multimodal Affective User Interface; (**b**) Framework of AVRS; (**c**) VR scenes cut show.

Combining visual and auditory senses, movie stimulation has much progress. In the work of [15], at first, the authors built a Multimodal Affective User Interface (Figure 4a) to help to gather users’ emotion-related data and their emotions. After conducting a pilot panel study with movie scenes to determine some high-quality films, the authors finally chose 21 movie clips to arouse anger, sadness, amusement, disgust, fear and surprise. The film names were given in this paper. Music video also plays an important role in emotion stimulation. In [16], 32 subjects watched 40 one-minute long music videos. These video clips were selected using a subjective emotion evaluation interface online from 120 stimuli one-minute videos. The authors used an affective highlighting algorithm to extract a one-minute highlight clip from each of the 120 original music videos. Half of the videos were chosen manually, while the other half were selected via affective tags from the website. 

Zhang et al. proposed a novel emotion evocation system called Affective Virtual Reality System (AVRS, Figure 4b, [17]), which was composed of eight emotive VR scenes (Figure 4c) and their three-dimensional emotion indexes that were evaluated by 100 subjects using the Self-Assessment Manikin (SAM). Color, sound and other features were extracted to create affective VR scenes. These features were selected from standard emotive picture, video and audio materials such as IADS and so forth. Emotions can be elicited more efficiently and accurately via AVRS according to this paper. A multiplayer first-person shooter computer game was used to induce emotion in [18]. Participants were required to bring their friends as their teammates. Through this game, subjects became more immersed in a virtual world and pay less attention to their environment. Moreover, a recall paradigm and autobiographical memories were used in [19,20] respectively.

## 3. Emotional Relevant Features of Physiological Signals

**Figure 5:**
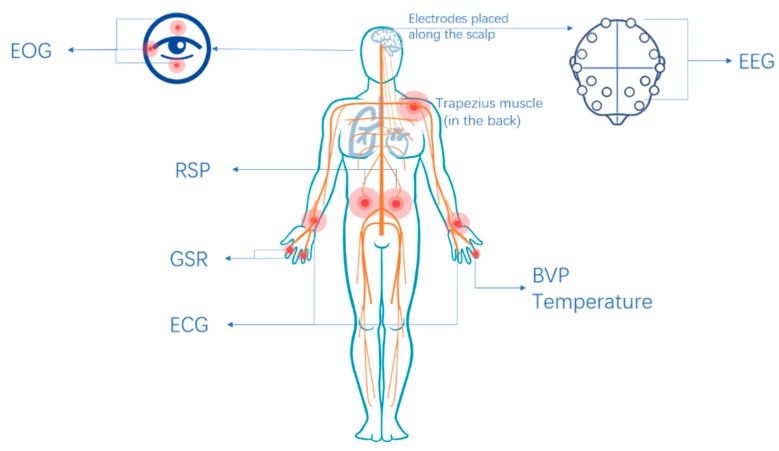
Position of the bio-sensors.

The position of the biosensors used is illustrated in Figure 5.

### 3.1. EEG

**Figure 6:**
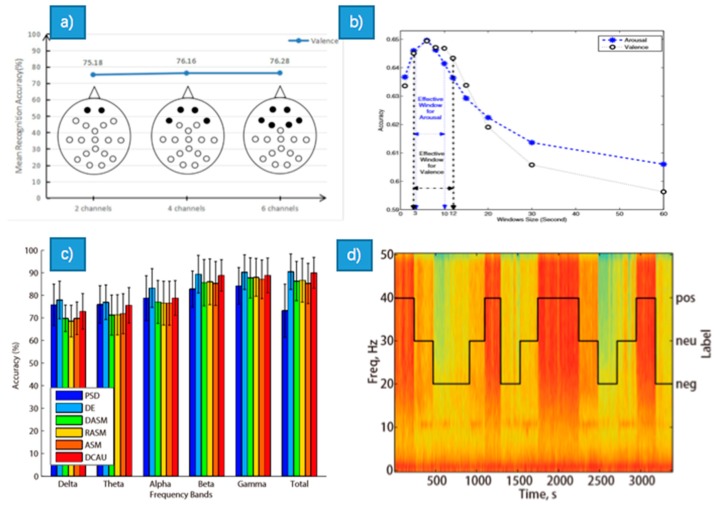
(**a**) Mean accuracy of different channels; (**b**) The performance of different windows sizes; (**c**) The average accuracies of GELM; (**d**) Spectrogram shows different patterns with different emotions.

In the reference [21], the authors provided a method for emotion recognition using only two channels of frontal EEG signals at Fp1 and Fp2. It took advantages of spatial, frequency and asymmetry characteristics of EEG signals (Figure 6a). The experiment using a GBDT (Gradient boosting Decision Tree) classifier validated the effectiveness of the method, where the maximum and mean classification accuracy were 76.34% and 75.18% respectively. 

A novel real-time emotion recognition algorithm was proposed [22] based on the most stable features such as Fractal Dimension (FD), five statistics features (standard deviation, mean of absolute values of the first differences, mean of absolute values of the first differences of normalized EEG, mean of absolute values of the second differences, mean of the absolute values of the second differences of the normalized EEG), 1st order Higher Order Crossings (HOC) and four band power features (alpha power, theta power, beta power, theta/beta ratio). This algorithm is subject-dependent which needs just one training for a new subject and it has the accuracy of 49.63% for 4 emotions classification, 71.75% for two emotions classification, and 73.10% for positive/negative emotions classification. With the adequate accuracy, the training results can be used in real-time emotion recognition applications without re-training.

In the work of [23], the author proposed a novel model for multi-subject emotion classification. The basic idea is to extract the high-level features through the deep learning model and transform traditional subject-independent recognition tasks into multi-subject recognition tasks. They used the Convolutional Neural Network (CNN) for feature abstraction, which can automatically abstract the correlation information between multi-channels to construct more discriminatory abstract features, namely, high-level features. And the average results accuracy of the 32 subjects was 87.27%.

The features with DWT were used in [24] with varying window widths (1~60 s) and the entropy was calculated of the detail coefficients corresponding to the alpha, beta, and gamma bands. Using the SVM classification, the classification accuracy in arousal can be up to 65.33% using a window length of 3–10 s, while 65.13% in valence using a window length of 3–12 s. The conclusion is that the information regarding these emotions may be appropriately localized at 3–12 s time segments. In reference [25], the authors systematically evaluated the performance of six popular features: power spectral density (PSD), differential entropy (DE), differential asymmetry (DASM), rational asymmetry (RASM), asymmetry (ASM) and differential caudality (DCAU) features from EEG. Specifically, PSD was computed using Short Time Fourier Transform (STFT); DE was equivalent to the logarithmic power spectral density for a fixed length EEG sequence; DASM and RASM features were the differences and ratios between the DE features of hemispheric asymmetry electrodes; ASM features were the direct concatenation of DASM and RASM features. The results showed that GELM with DE features outperforms other methods, which achieved the accuracy of 69.67% in DEAP dataset and 91.07% in SEED dataset. The average accuracies of GELM using different features obtained from five frequency bands are given in the Figure 6c. And Figure 6d shows that the spectrogram has different patterns as different emtions elicited. In [26], the authors employed Hjorth Parameters for feature extraction which was a statistical method available in time and frequency domain. The Hjorth parameters were defined as normalized slope descriptors (NSDs) which contained activity, mobility and complexity. Using SVM and KNN as the classifiers, the highest classification result of four emotions was 61%. Comparatively, their results showed that the KNN was always better than SVM.

A new framework which consists of a linear EEG mixing model and an emotion timing model was proposed (Figure 6b) [27]. Specially, the context correlations of the EEG feature sequences were used to improve the recognition accuracy. The linear EEG mixing model based on SAE (Stack AutoEncoder) was used for EEG source signals decomposition and for EEG channel correlations extraction, which reduced the time in feature extraction and improved the emotion recognition performance. The LSTM (Long-Short-Term-Memory Recurrent Neural Networks) was used to simulate the emotion timing model, which can also explore the temporal correlations in EEG feature sequences. The results showed that the mean accuracy of emotion recognition achieved 81.10% in valence and 74.38% in arousal, and the effectiveness of the SAE + LSTM framework was validated.

The authors of [28,29,30] introduced the changes of several typical EEG features reflecting emotional responses. The study indicated that the PSD of alpha wave of happiness and amusement was larger than that of fear, and PSD of gamma wave of happiness was also greater than that of fear. However, there was not obvious difference in PSD of beta wave [31,32] among various emotions. As for DE [33], in positive emotions it was generally higher than in negative ones. The correlations between EEG features and emotions are summarized in Table 1. In general, using electroencephalography to recognize different emotions is a powerful and popular method, as the signals are able to be processed, and the changes of them are evident. It is advised to put electroencephalography as the major category in emotion recognition.

### 3.2. ECG

**Figure 7:**
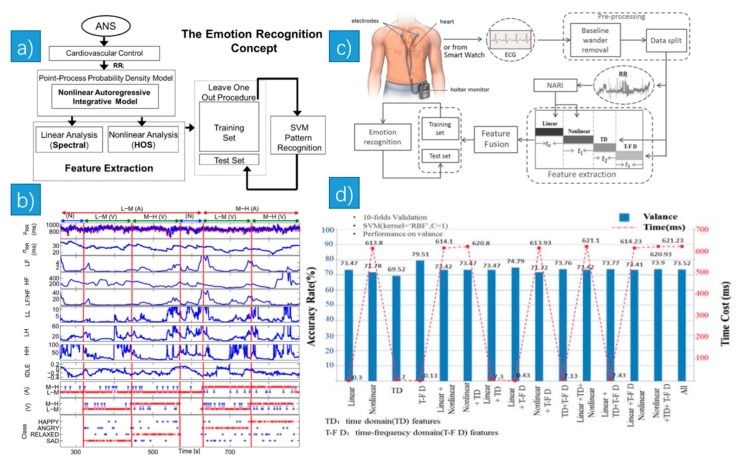
(**a**) Logical scheme of the overall short-time emotion recognition concept; (**b**) Instantaneous tracking of the HR V indices computed from a representative subject using the proposed NARI model during the passive emotional elicitation (two neutral sessions alternated to a L-M and a M-H arousal session); (**c**) Diagram of the proposed method; (**d**) Experimental results.

In the work of [34], the authors used a short-time emotion recognition concept (Figure 7a). They described five linear and eleven nonlinear features. The linear features were the mean and standard deviation (STD) of the (Inverse Gaussian) IG probability distribution, the power in the low frequency (LF) (0.04–0.15 Hz) and the high frequency (HF) (0.15–0.4 Hz) band, and the LF/HF ratio. The nonlinear features included the features from instantaneous bispectral analysis, the mean and STD of the bispectral invariants, mean magnitude, phase entropy, normalized bispectral entropy, normalized bispectral squared entropy, sum of logarithmic bispectral amplitudes, and nonlinear sympatho-vagal interactions. Some HRV indices extracted from a representative subject is shown in Figure 7b.

Two kinds of feature set were extracted in [35]. One was the standard feature set, including time domain features and frequency domain features. The other was the nonlinear feature set. 89 standard features and 36 nonlinear features were extracted from the signals. In the reference [36], the authors extracted the Hilbert instantaneous frequency and local oscillation from Intrinsic Mode Functions (IMFs) after applying Empirical Mode Decomposition (EMD). The study [37] used three methods to analyze the heart rate varibility (HRV) including time, frequency domain analysis methods and statistics analysis methods. The time domain features included mean and STD of RR intervals, coefficient of variation of RR intervals, STD of the successive differences of the RR intervals. The frequency domain features included LF power, HF power and the ratio of LF/HF. The statistic features included kurtosis coefficient, skewness value and the entropy.

**Figure 8:**
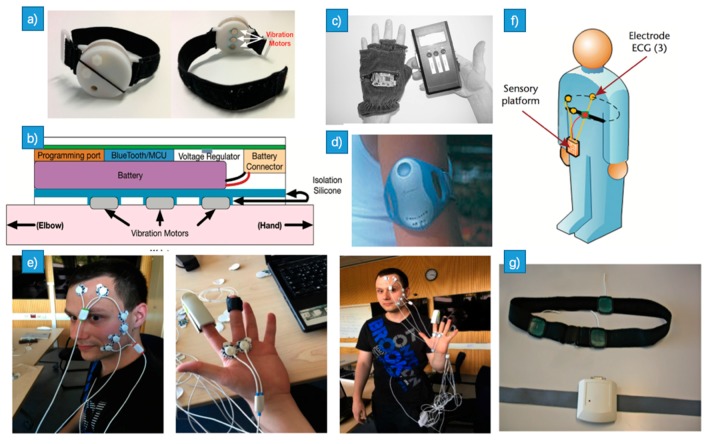
(**a**) The Emotion Check device; (**b**) Diagram describing the components of the Emotion Check device; (**c**) Prototype of glove with sensor unit; (**d**) Body Media Sense Wear Armband; (**e**) Left: The physiological measures of EMG and EDA. Middle: The physiological measures of EEG, BVP and TMP. Right: The physiological measures of physiological sensors in the experiments; (**g**) Illustration of R-TIPS. This platform allows wireless monitoring of cardiac signals. It consists of a transmitter system and three sensors; (**f**) The transmitter system is placed on the participant’s hip, and the sensors are placed below right breast, on the right side, and on the back.

In [38], the authors used various feature sets extracted from one-channel ECG signal to detect negative emotion. The diagram of the method is shown in Figure 7c. They extracted 28 features in total, including 7 linear-derived features, 10 nonlinear-derived features, 4 time domain features (TD) and 6 time-frequency domain features (T-F D). 5 classifiers including SVM, KNN, RF, DT and GBDT were also compared. Among all these combinations, the best result was achieved by using only 6 T-F D features with SVM (Figure 7d), which showed the best accuracy rate of 79.51% and the lowest time cost of 0.13 ms. In the study of [39], the authors collect EMG, EDA, ECG and other signals from 8 participants using the Biosignalplux research kit, which is a wireless real-time bio-signal acquisition unit with a series of physiological sensors. The positions of the biosensors they used are illustrated in Figure 8e. Among SVM, KNN, Decision Tree (DT) they used, DT gave the highest accuracy with the ST, EDA, EMG signals. In [40], based on an interactive virtual reality game, the authors proposed a novel GT-system, which allows the real-time monitoring and registration of psychological signals. An electronic platform (R-TIPS4) was designed to capture the ECG signal (Figure 8g). The position of R-TIPS4 was shown in Figure 8f.

The authors of [41] explored the changes of several main cardiovascular features in emotional responses. The response to anger induced increased heart rate (HR), increased diastolic blood pressure (DBP), and systolic blood pressure (SBP), and increased total peripheral resistance (TPR) [42]. Other studies also found increased DBP, SBP, and TPR in the same condition [43], as well as increased HR, DBP, SBP, and unchanged TPR [42]. As for happiness, it could be linked with increased HR [44] or unchanged HR [45], decreased heart rate variability (HRV) [46], and so on. Concerning fear, kinds of studies reported increased HR, decreased finger temperature (FT) [47], decreased finger pulse amplitude (FPA), decreased finger pulse transit time (FPTT) [48], decreased ear–pulse transit time (EPTT), and increased SBP and DBP [47]. Concerning amusement, it could be characterized by increased HRV, unchanged low frequency/high frequency ratio (LF/HF) [49], increased pre-ejection period (PEP), and decreased cardiac output (CO) [50]. Decreased FPA, FPTT, EPTT, and FT [51], increased TPR [50], decreased FPA, and unchanged FT [46] have also been reported. The relationship between cardiovascular feature changes and emotions are summarized in the Table 1. In conclusion, although some features might have different changes in the same emotion, to some extent, putting all features in overall consideration can eliminate the difference. The typical features in cardiovascular system can describe different emotions in a more objective and visual way, since they provide a few features that are able to be measured and analyzed.

### 3.3. HR

A novel and robust system was proposed which can collect emotion-related physiological data over a long period of time [52]. Using wireless transmission technology, this system will not restrict users’ behavior and can extract ideal physiological data in accord with the real environment. It can extract users’ skin temperature (ST), skin conductivity (SC), environmental temperature (ET) and their heart rate (HR). The ST, SC ET sensors were integrated into a glove (Figure 8c) while the HR sensor used was a conventional chest belt. In the study of [53], subjects were required to watch a 45-min slide show while their galvanic skin response (GSR), heart rate (HR), and temperature were measured using Body Media Sense Wear Armband (Figure 8d). These physiological data were normalized and four features including minimum, maximum, mean, and variance of them were extracted. The three algorithms, KNN, DFA, and MBP they chose could recognize emotions with the accuracy of 72.3%, 75.0% and 84.1% respectively. The researchers had built the Emotion Check [54], which is a wearable device that can detect users’ heart rate and regulate their anxiety via false heart rate feedback. Figure 8a,b show this device and its components respectively.

### 3.4. GSR

In the work of [55], the authors used galvanic skin response (GSR), fingertip blood oxygen saturation (OXY) and heart rate (HR) as input signals to recognize five emotions by random forests. They calculated 12 conventional GSR features, including the mean and STD of GSR, the average and root mean square of 1st differences deviation of GSR, the number, average amplitude, average duration and maximum amplitude of skin conductance response (SCR), the mean of the absolute values of 1st differences of the raw GSR, the mean of the GSR filtered by a Hanning window and the mean of the absolute values of 1st and 2nd differences of the normalized GSR. The noisy fluctuations were eliminated by using a Hanning window filter. Also, the fluctuations of GSR and first deviation of GSR (FD_GSR) in different time scales were applied as affective features, which was called LSD. Finally, a total 43 GSR and FD_GSR features were obtained and yielded an overall accuracy rate of 74%. In the work of [56], the authors chose GSR, HR and RSP as input signals to classify negative emotions from neutral by Fuzzy-Adaptive Resonance Theory and yielded a total accuracy rate of 94%. The GSR-dif extracted from GSR was defined as: GSR-dif = (GSR-max) − (GSR-base).

Six emotions were recognized from GSR signals by Fisher classifier [57]. 30 statistical features were extracted such as range, maximum and minimum of the GSR. Then immune hybrid Particle Swarm Optimization (IH-PSO) was used to reduce the features. The average verifying recognition rates of surprise, fear, disgust, grief, happy and angry respectively reached 78.72%, 73.37%, 70.48%, 62.65%, 62.52% and 44.93%. In [58], the author combined ECG and GSR signals to recognize emotions among happy, sad and neutral. The PSD features of ECG and GSR were extracted. The performance of the emotional state classification for happy-sad, sad-neutral and happy-neutral emotions was 93.32%, 91.42% and 90.12% respectively. 

**Figure 9:**
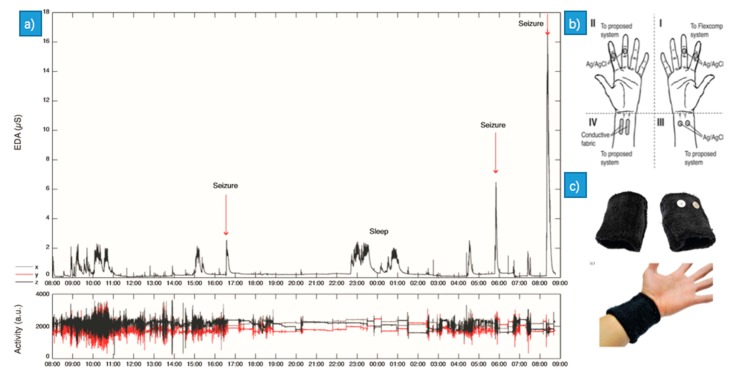
(**a**) Monitoring of epileptic seizures using EDA; (**b**,**c**) Wearable GSR sensor.

A novel solution [59] was presented to enable comfortable long-term assessment of EDA (the same as GSR) in the form of a wearable and fully integrated EDA sensor (Figure 8e,f). The novelty of their work consists of the use of the dorsal forearms as recording sites which performed better than the traditional palmar recording sites, as well as the investigation of how the choice of electrode material affects performance by comparing the use of conductive fabric electrodes to standard Ag/AgCl electrodes. Finally, they presented a one-week recording of EDA during daily activity, which is the first demonstration of long-term, continuous EDA assessment outside a laboratory setting. In [60], the author used the wearable EDA sensor suitable for long-term monitoring to monitor sympathetic nervous system activity during epileptic seizures. It was based on the fact that epileptic seizures induce a surge in EDA. They found that the change in EDA amplitude (Figure 9) was significantly higher after generalized tonic-clonic seizures (GTCS) seizures compared to CPS.

The author of [41] introduced changes in several main electrodermal features when reflecting emotional responses. In anger responses, this included increased SCR [61]; increased, non-specific skin conductance response rate (nSRR); and increased SCL [62]. As for happiness, it can be characterized by increased SCL [63] and increased nSRR [64]. Some studies also reported unchanged SCL [65] or decreased SCL [66]. Concerning fear, some studies reported increased SCR [67] ,increased nSRR [68] and increased SCL [69]. Concerning amusement, it can be characterized by increased SCR [70], increased nSRR, and increased SCL [71]. The electrodermal feature changes under emotions are summarized in Table 1. In short, according to these findings, the features in Electrodermal System almost have an identical trend for different emotions. There can be an auxiliary mean when using other physiological features to recognize the emotion.

### 3.5. RSP

Researchers of [72] used particle swarm optimization (PSO) of synergetic neural classifier for emotion recognition with signals of EMG, ECG, SC, RSP. The breathing rate, amplitude and other typical statistical features as mean and STD are extracted from the RSP. The total classification rate was 86% of four signals for four emotions. In [73], the authors extracted features from ECG and RSP to recognize emotions. The followings are features extracted from respiration and the respiratory sinus arrhythmia (RSA): the respiratory instantaneous frequency and amplitude, the amplitude ratio of the RSA to the respiratory oscillation, the difference between the RSA and the respiratory frequencies, the phase difference of the RSA and the respiration, the slope of this phase difference and its STD. His experiment showed that using the feature of the slope of the phase difference of the RSA and the respiration got the best correct classification rate of 74% for valence, 74% for arousal and 76% for liking.

A new methodology [35] was reported using ECG, EDR and RSP. He extracted both the standard and nonlinear features. The standard features included maximum and minimum respiration rate, spectral power and mean and standard deviation of the first and second derivative, High Order Statistics (HOS) as the third order statistics, the fourth order statistics and the standard error of the mean (SEM). Recurrence Quantification Analysis (RQA), Deterministic Chaos (DC), Detrended Fluctuation Analysis (DFA) were used to extract the nonlinear features. The experiment got a recognition rate of 90% for arousal and 92% for valence by using nonlinear features. Another new methodology [74] named Respiration quasi-Homogeneity Segmentation (RHS) was used to extract Emotion Elicited Segments (EESs) where the emotion state could be reliably determined. The new method yielded a classification rate of 88% for five emotions.

In the reference [41], the author introduced the changes of several main respiratory features in reflecting emotional responses. In anger responses, it included unchanged [44] or increased respiration rate (RR), increased functional residual capacity (FRC) [75], shortened inspiratory time (Ti) and expiratory time (Te), increased post-inspiratory pause time (Pi) [76], decreased inspiratory/expiratory ratio (I/E-ratio) [77]. As for happiness, it could be characterized by increased RR [78] or unchanged RR [46], decreased Ti and Te, decreased post-expiratory pause time (Pe) [78], increased Pi and FRC [44]. Concerning fear, various of studies reported increased RR, and either both decreased Ti and Te [79], or primarily increased Pi, decreased Te and unchanged Ti [44]. About amusement, it could be characterized by increased RR [51], decreased Ti and tidal volume (Vt) [76]. The respiratory feature changes versus emotions are shown in Table 1. Using respiratory features to recognize different emotions is also a powerful method since the change of features is apparent and the measures of each features is accessible. It is advised to add the respiratory features to enhance the accuracy of the recognition.

### 3.6. EMG

In the work of [80], the authors adopted EMG, RSP, skin temperature (SKT), heart rate (HR), skin conductance (SKC) and blood volume pulse (BVP) as input signals to classify the emotions. The features extracted from the EMG are temporal and frequency parameters. Temporal parameters are mean, STD, mean of the absolute values of the first and the second difference (MAFD, MASD), distance, etc. The frequency parameters are the mean and the STD of the spectral coherence function. It attained a recognition rate of 85% for different emotions. Reference work [81] used ECG, EMG, SGR as signals to classify eight emotions. A 21-feature set was extracted from facial EMG, including mean, median, STD, maxima, minima, the first and the second derivatives of the preprocessed signal and the transformation.

Facial EMG can be used to recognized emotions [82]. The extracted features were higher order statistics (HOS) and six independent statistical parameters. The HOS feature included Skewness (degree of asymmetry of the distribution of its mean) and Kurtosis (the relative heaviness of the tail of the distribution about the normal distribution). The statistical features included the normalized signals, STD of the raw signal, mean of absolute value of the first and the second difference of raw and normalized signals. A total recognition rate of 69.5% was reached for six different emotions. Surface EMG was used as signals to classify four emotions [83]. The authors decomposed signals by discrete wavelet transform (DWT) to select maxima and minima of the wavelet coefficients and got a total recognition rate of 75% by BP neural network with mere EMG. Study [84] used the same features to classify four emotions and got a recognition rate of 85% by support vector machine (SVM). Another study [7] for emotion recognition is proposed based on the EMG. The following are features used in the study: mean, median, STD, minimum, maximum, minimum rate, maximum rate of the preprocessed signals. The same features are extracted from the first and the second difference of the signals. The study got a total recognition rate of 78.1% for six emotions classification. The study [85] decomposed EMG by DWT under four frequency ranges. The statistical features were extracted from above wavelet coefficients. The proposed method yielded a recognition rate of 88%.

## 4. Methodology

**Figure 10:**
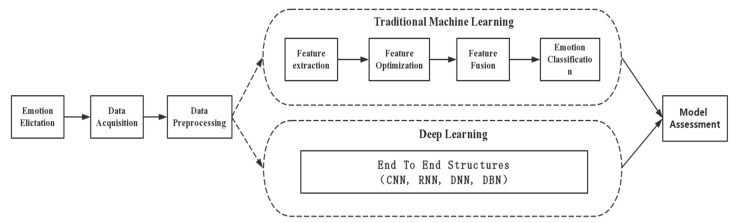
Emotion recognition process using physiological signals under target emotion stimulation.

This section mainly focuses on the methodology of physiological signal-based emotion recognition, which can be divided into two major categories. One is using the traditional machine learning methods, which are also considered as model specific methods. They require carefully designed hand-crafted features and feature optimization. The other is using the deep learning methods, which are model free methods. They can learn the inherent principle of the data and extract features automatically. The whole emotion recognition framework is shown in Figure 10. Signal preprocessing, which is included both in traditional methods and deep learning methods, is adopted to eliminate the noise effects caused by the crosstalk, measuring instruments, electromagnetic interferences, etc. For the traditional machine learning methods, it is very necessary to explore the emotion-specific characteristics from the original signals and select the most important features to enhance the recognition model performance. After feature optimization and fusion, classifiers which are capable of classifying the selected features are utilized. Unlike the traditional methods, deep learning methods no longer require manual features, which eliminate challenging feature engineering stages of the traditional methods.

### 4.1. Preprocessing

It is extremely necessary to eliminate the noise effects at the very early stage of emotion recognition by preprocessing, due to the complex and subjective nature of raw physiological signals and the sensitivity to noises from crosstalk, measuring instruments, electromagnetic interferences, and the movement artifacts.

Filtering: The low-pass FIR filter is commonly used in removing noises. In the work of [82], the signal crosstalk was removed by means of a notch filter, after which a smooth process was taken to avoid the influence of the signal crosstalk by 128-point moving average filter (MAF). The same filter was used in [35] to minimize the baseline and artifact errors from RSP. High pass filters were adopted in [10] with cut-off frequencies of 0.1 Hz and 4 Hz in processing RSP and ECG respectively to eliminate the baseline wander.

DWT**:** In the studies of [86,87], DWT was used to reduce noises of the physiological signals. As the orthogonal WT of a white noise is a white noise, according to the different propagation characteristics of the signals and the noises at each scale of the wavelet transform, the modulus maximum point generated by the noise can be removed, and the modulus maximum point corresponding to the signals can be retained, then the wavelet coefficients can be reconstructed by the residual modulus maxima to restore the signals.

ICA: Independent component analysis (ICA) was used to extract and remove respiration sinus arrhythmias (RSA) from ECG [35], where it decomposed the raw signals into statistically independent components, and the artifact components can be removed by observing with eyes, which required some expertise. When there were limited signals, some cortical activities might be considered as artifact component. An artifacts removal method based on hybrid ICA-WT (wavelet transform) was proposed for EEG to solve the problem [88], which could significantly improve the recognition performance compared to the regular ICA algorithm. In [89], the authors compared three denoising algorithms, namely principal component analysis (PCA), ICA and multiscale principal component analysis (MSPCA), where the overall accuracies were 78.5%, 84.72%, 99.94% for PCA, ICA, MSPCA respectively.

EMD: Empirical mode decomposition (EMD) can be used to remove the eye-blink form EEG. The EEG signals mixed with eye-blink was decomposed into a series of intrinsic mode functions (IMFs) by EMD [90], where some IMFs represented the eye-blink. A cross-correlation algorithm was then proposed with a suitable template extracted from the contaminated segment of EEG, which caused less distortion to the brain signals and efficiently suppressed the eye-blink artifacts.

In general, for the obvious abnormal signals, such as the exfoliation of electrodes in the collection of signals, or the loss of signals caused by unintentional extrusion of the subjects, the artifact components can be removed through visual observation, which requires some expertise. For the interference signals contained in the normal original signals, different methods (filtering, DWT, ICA, EMD) are needed to reduce the noise according to the characteristics of the time domain and frequency domain of different physiological signals and different sources of interferences.

In particular, for filters, different types of low-pass filters such as Elliptic filters, Adaptive filters, Butterworth filters etc., are used to preprocess the ECG and EMG signals. Smoothing filters are often used to pre-process the raw GSR signals.

### 4.2. Traditional Machine Laerning Methods (Model-Specific Methods)

In the traditional machine learning methods, there are processes including feature extraction, optimization, fusion and classification.

#### 4.2.1. Feature Extraction

Feature extraction plays a very important role in the emotion recognition model. Here several major feature extraction methods have been surveyed, like DWT, ICA, E MD, FFT, autoencoder, etc.

##### FFT and STFT

It’s important to extract the most prominent statistical features for emotion recognition. The physiological signals like EEG are complex and non-stationary, under which conditions some statistical features like power spectral density (PSD) and spectral entropy (SE) are widely-known applicable features in emotion recognition. Therefore, FFT was adopted to calculate the spectrogram of the EEG channels [91,92]. Due to the shortcoming that the FFT can’t deal with the non-stationary signal, STFT was proposed: By decomposing the entire signals into numerous equal-length pieces, each small piece can be approximately stationary, hence FFT can be applicable. In the work of [29,93], a 512-point STFT was presented to extract spectrogram from 30 channels of the EEG.

##### WT

For non-stationary signals, a small window suits high frequency and a large window suits low frequency. While the window of the STFT is fixed, which limits its application. Wavelet transform provides an unfixed ‘time-frequency’ window and is able to analyze the time-space frequency locally, therefore is suitable for decomposing the physiological signals into various time and frequency scales. The basis functions WT(a, b) are described as below:

(1) $$WT(a,b)=\frac{1}{\sqrt{a}}\int_{-\infty }^{+\infty }f(t)*\varphi (\frac{t-b}{a})dt\;a,b\in R,a>0$$

where *a* is the scale parameter, *b* refers to the translation parameter and the φ is the mother wavelet. The performance of the WT is affected mostly by the mother wavelet. The low scale is in accordance with the high frequency of the signal and the high scale is in accordance with the low frequency of the signal. There are several common mother wavelets, such as Haar, Daubechies, Coif, Bior wavelets, etc. The coefficients after the WT can be used to reproduce the original signal. Db-4 wavelet was applied to conduct continuous WT (CWT) for the EEG signal [94]. In the work of [95], CWT with Db-4, Morlet, Symlet2, Haar were used for EMG, ECG, RSP, SC respectively. DWT with Db-5 wavelet was applied to analyze the high frequency coefficients at each level of five EEG frequency bands which included delta, theta, alpha, beta and gamma [96]. DWT with Db-5 wavelet for six levels was used for analyzing EMG [97]. The reference work [98] decomposed the EEG signal with Db-4 wavelet into five levels. Several mother wavelets were tested and the Symlets6 outperformed others for 4 levels [99].

##### EMD

**Figure 11:**
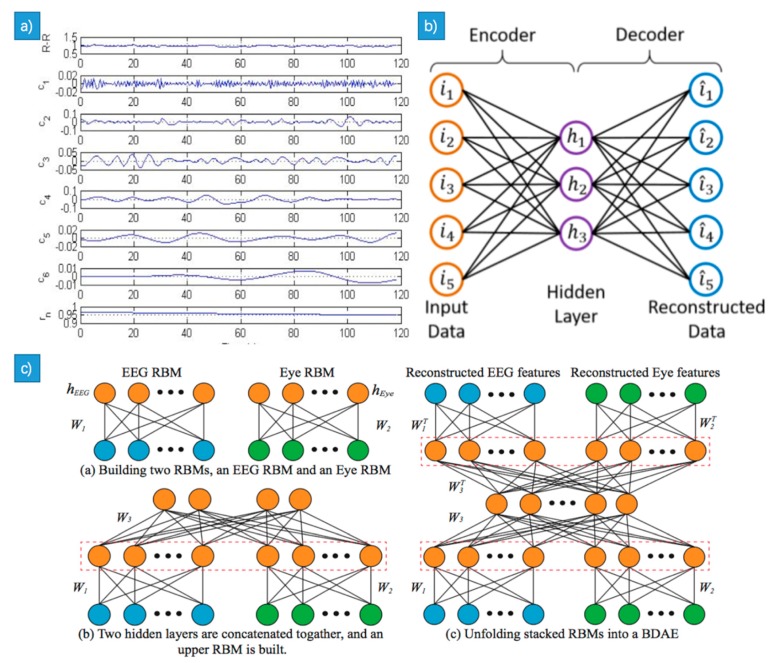
(**a**) The decomposition of R-R interval signal (emotion of sadness); (**b**) The structure of Autoencoder; (**c**) The structure of Bimodal Deep AutoEncoder.

EMD is a powerful tool that decomposes the signals according to time scale characteristics of the signal itself without any pre-set base functions. The EMD method can be applied in theory to the decomposition of any type of signal, and thus has a very obvious advantage in dealing with non-stationary and nonlinear signals with high signal-to-noise ratio. Hilbert-Huang transform method (HHT) based on the EMD was tried, where each signal was decomposed into IMFs components using EMD (see Figure 11a) [100]. Four features were extracted from each IMF and were combined together from different number of IMFs. EEMD was proposed to solve the signal aliasing problem when applying EMD, which added the white Gaussian noise to the input signals and produce the average weight after a couple of EMD decomposition [87]. It’s still different between the IMFs decomposed from signals with and without high-frequency noises even if the signals look similar, due to decomposition of uncertainty. A method named the bivariate extension of EMD (BEMD) was proposed in [36] for ECG based emotion recognition, who compounded an ECG synthesis signal with the input ECG signal. The synthetic signals which were without noise and can be used as a decomposition guide.

##### Autoencoder

Autoencoder is an unsupervised algorithm based on the BP algorithm, which contains an input layer, one or more hidden layers and an output layer (as can be seen in Figure 11b). The dimension of the input layer is equal to that of the output layer, so that it was called ‘encoder network’ (EN) from input layer to the hidden layer and ‘decoder network’ (DN) from hidden network to output layer. The autoencoder works as below: At first the weights of the EN and the DN are initiated. Then the autoencoder is trained according to the principle that minimizes the error between the original data and the reconstructed data. It is easy to get the desired gradient value by passing the chaining method of the DN and passing the CN using the backward propagation error derivation and adjusting the weighted value of the autoencoder to the optimal one. The high level of the feature extraction of the bimodal deep autoencoder (BDAE, as can be seen in Figure 11c) is effective for affective recognition [101], where two restricted Boltzmann machine (RBM) of EEG and eye movements were built. The shared features extracted from the BDAE were then sent to the SVM. 

Many physiological signals are non-stationary and chaotic. To extract information from non-stationarity in physiological signals and reduce the impact of non-stationary characteristics on subsequent processing, FFT and STFT are adopted to obtain the features in frequency domain. The time window length and type of STFT exhibit significant influences on the transformation result, which are specific to different signals and different methods.

In addition to the above-mentioned methods in frequency domain and time domain, the signal decomposition algorithm in spatial domain is also popularly applied in EEG signal processing. The feature based on spatial domain is usually used to separate EEG signals from different brain regions. Studies approve that combined features of time domain, frequency domain, and time-frequency domain could be closer to the ground truth when emotions change.

#### 4.2.2. Feature Optimization

There might be a quantity of features after the feature extraction process, some of which might be irrelevant, and there are probably correlations between the features. It may easily lead to the following consequences when there are a number of redundant features: (1) it would take a long time to analyze the features and train the model; (2) it is easy to cause overfitting problems and a poor ability of generalization which leads a low recognition rate; (3) it is easy to encounter the problem of sparse features, also known as ‘curse of dimensionality’, which results in the decrease of the model performance. Therefore, it is very necessary to conduct feature optimization.

ReliefF algorithm was used due to its effectiveness and simplicity of computation [102]. The key point of RelifF is to evaluate the ability of features to distinguish from examples which are near to each other. In the work of [103], the authors used maximum relevance minimum redundancy (mRMR) to further select features. The next feature to choose was calculated by the following formula:

(2) $$\underset{x_{j}\in X-S_{k}}{Max}\left[I(x_{j};y)-\frac{1}{k}\sum_{x_{i}\in S_{k}}I(x_{j};x_{i})\right]$$

where $I(x_{j};y)$ is the mutual information between the feature and the specific label, $\frac{1}{k}\sum_{x_{i}\in S_{k}}I(x_{j};x_{i})$ is the average mutual information between two features. $S_{k}$ denotes the chosen set of k features. Due to the uncertainty of the results of using the mRMR, the authors applied the algorithm for each feature from one to the last dimension.

Sequential backward selection (SBS) and sequential forward selection (SFS) were applied to select features [72]. SBS starts with a full set of features and iteratively removes the useless features, while SFS starts with an empty set and adds the feature to the feature set which improves the performance of the classifier. After applying the feature selection algorithm, the recognition rate increased almost 10% from 71% to above 80%. The SBS and tabu search (TS) were used in the study to select features [86], where it reduced almost half of the features when using TS only and got an average recognition rate of 75.8%. While using both TS and SBS, it again reduced lots of features and got a higher recognition rate of 78.1%. TS was applied to reduce the features from 193 to only 4 [104], and it still got a recognition rate of 82% for 4 affective states.

In [35], the authors used PCA to reduce features, which could project the high dimensional data to a low dimensional space with a minimal loss of information. Combined with the Quadratic Discriminant Classifier (QDC), it got a recognition rate of 90.36%, 92.29% for valence and arousal of five classes respectively. The kernel PCA quantity was conducted to extract features to form the spectral powers of the EEG [105], that can compute higher order statistics among more than two spectral powers compared with the common PCA. The genetic algorithm (GA) was used to select IMFs after EMD process, where the selected IMFs were used either to reconstruct the new input signal or to provide separate features that represented the oscillation coexisting in the original signals, which was named as hybrid adaptive filtering (HAF) by the authors [106].

There are several feature selection algorithms except these mentioned above. In general, some algorithms reduce the dimensionality by taking out some redundant or irrelevant features (ReliefF, SFS, SBS, TS), and other algorthms transform the original one into a new set of features (PCA, ICA). The performance of the feature selection algorithms depends on the classifier and the dataset, and the universal feature selection algorithms do not exist.

#### 4.2.3. Feature Fusion

The techniques of feature fusion can be divided into three categories: early, intermediate and late fusion. In the early fusion (feature level fusion), the features selected from the signals are combined together in a single set before sending them to the classifier. The intermediate fusion can cope with the imperfect data reliably. Late fusion, which is also called decision level fusion, represents that the final result is voted by the results generated by several classifiers. The early fusion and the late fusion are most widely used in integrating signals.

##### Early Fusion

When different features are integrated into a single feature set before classifying process, the fusion is called as early fusion. In the early fusion, the single level recognition process in some specific modes affects the course of the remaining pattern of the recognition process. Ultimately, this fusion is found to be more suitable for highly timely synchronized in the input mode. Audio-visual integration might be the most suitable example of the early fusion in which audio and visual feature vectors are simply connected to obtain a combined audio-visual vector.

**Figure 12:**
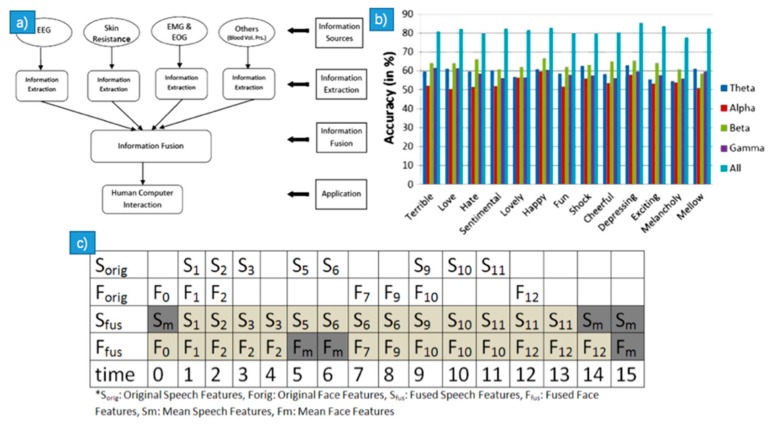
(**a**) Typical framework of multimodal information fusion; (**b**) SVM results for different emotions with EEG frequency band; (**c**) Demo of the proposed feature level fusion. A feature vector created at any time step is valid for the next two steps.

The early fusion was employed in the study of [96], whose framework of information fusion can be seen in Figure 12a. The author proposed a multimodal method to fuse the energy-based features extracted from the 32-channel EEG signals and then the combined feature vector was trained by the SVM and got a recognition rate of 81.45% for thirteen emotions classification (Figure 12b). In reference [107], the authors applied the HHT on ECG to recognize human emotions, where the features were extracted through the process of fission and fusion. The features extracted from IMFs were combined into a feature vector. From fission process, the raw signals were decomposed into several IMFs, then the instantaneous amplitude and instantaneous frequency were calculated from IMFs. The fusion process merged features extracted from the fission process. In the study of [108], the authors proposed an asynchronous feature level fusion method to create a unified mixed feature space (Figure 12c). The target space can be used for clustering or classification of multimedia content. They used the proposed method to identify the basic emotional state of verbal rhythms and facial expressions.

##### Intermediate Fusion

The shortcoming of early fusion is its inability to cope with imperfect data as well as the asynchrony problem. The intermediate fusion can deal with these problems. One way to overcome these problems is to consider the characteristics of the corresponding flow in various time instances. Thus, by comparing the previously observed instances with the current data of some observing channels, some statistical predictions of certain probable probabilities for erroneous instances can be made. Models like hidden Markov model (HMM), Bayesian network (BN) are useful to deal with the situation mentioned before. A BN was built to fuse the features from EEG and ECG to recognize emotions [109].

##### Late Fusion

The late fusion typically uses separate classifiers that can be trained separately. The final classification decision is made by combining the outputs of each single classifier. The identification of the correspondence between the channels is performed only in the integration step. Since the input signals can be recognized separately, there is not so much necessary to put them together at the same time. The authors of reference [110] used a hierarchical classifier to recognize emotions in the outdoor from video and audio. They extracted SIFT, LBP-TOP, PHOG, LPQ-TOP and audio features and then trained in several SVMs. Finally, they combined the results from the SVMs. The method yielded a recognition rate of 47.17% and was better than the baseline recognition rate. In [111], the authors put forward a framework for emotion recognition based on the weighted fusion of the basic classifiers. They developed three basic SVMs for power spectrum, high fractal dimension and Lempel-ziv complexity respectively. The results of the basic SVMs were integrated by weighted fusion which was based on the classifier confidence estimation for each class.

#### 4.2.4. Classification

**Figure 13:**
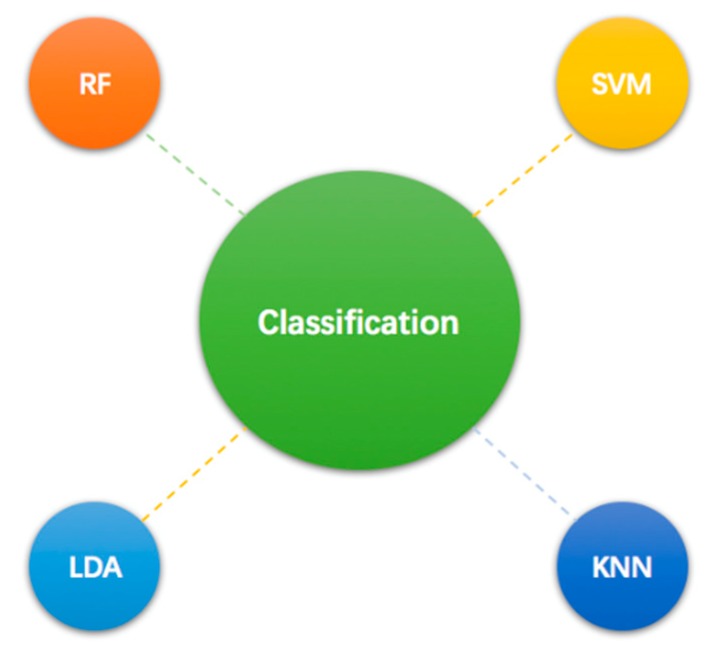
Classification models.

In emotion recognition, the major task is to assign the input signals to one of the given class sets. And there exists several classification models suitable for emotion recognition, including Linear Discriminant Analysis (LDA), Quadratic Discriminant Analysis (QDA), K-nearest neighbor (KNN), Random Forest (RF), particle swarm optimization (PSO), SVM, Probabilistic neural network (PNN), Deep Learning (DL), and Long-Short Term Memory (LSTM). Figure 13 shows 7 mainly used classification models.

SVM is a useful classifier for binary classification. Some data might not be correctly classified in low dimension space due to its non-linearity, and the sample space is mapped onto a high-dimensional space with kernel functions, in which the sample can be linearly separable. Kernel function can reduce the computational complexity brought by the dimension increasing [112]. As a lazy learning algorithm, KNN algorithm is a classifying method based on weights [113] and is relatively easy to understand and implement. However, KNN needs to store all training sets, which causes high complexities in time and space. The nonlinear classifiers, such as kernel SVM and KNN, calculate the decision boundary accurately, which may occur over fitting and affect the generalization ability. Compared with them, the generalization ability of LDA is better. As a linear classifier, LDA decides class membership by projecting the feature values to a new subspace [114]. For high-dimension data, the classification performance of RF and neural network algorithm is generally better. The main that difficulty lies in decision tree is overfitting; therefore, the classification result of RF [115] is usually decided by multiple decision trees to avoid the problem. CNN is an improvement to the traditional neural network, in which the weight sharing and the local connection can help to reduce the complexity of the network.

Various studies choose various physiological signals, feature sets, and stimulus sources, meaning that optimal classifier algorithms exist under various conditions. We can only discuss the optimal classifier under certain conditions. A more detailed discussion is made in the following section.

##### SVM

SVM is most widely used in physiological signal base emotion recognition. A framework called HAF-HOC was proposed [106]: Firstly, the EEG signal was input to the HAF section, where IMFs decomposed by the EMD were extracted and certain IMFs were selected by the Genetic Algorithm (GA), which could reconstruct the signals of EEG. This output of HAF was then used as input to the HOC section, where HOC-based analysis was performed resulting in the efficient extraction of the feature vectors which were finally sent to the SVM with a total recognition rate of 85%.

In the work of [58], the authors extracted the Welch’s PSD of ECG and Galvanic Skin response (GSR) signals and got a recognition rate of 91.62% by suing SVM. While in [101], features of EEG and eye signals were extracted by autoencoder. A linear SVM was then used which realized an average recognition rate of 91.49% for three emotion states. A model using Gaussian process latent variable models (GP-LVM) was proposed in [116], where a SVM was involved to train the latent space features and got a recognition rate of 88.33% and 90.56% for three levels of valence and arousal respectively. Nonlinear Autoregressive Integrative (NARI) was studied in [34] to extract features from HRV that yielded overall accuracy of 79.29% for four emotional states by SVM.

However, the regular SVM doesn’t work in the imbalanced dataset owing the same punishment weights for two classes and the optimal separating hyperplane (OSH) may tend to be the minority class. In the study of [105], an imbalanced support vector machine was used as the classifier to solve the imbalanced dataset problem, which increased the punishment weight to the minority class and decreased the punishment weight to the majority class. Never the less quasicon formal kernel for SVM was used to enhance the generalization ability of the classifier.

##### LDA

The LDA needs no additional parameters in the classification process. A limitation of the LDA is that the scattering matrices of the objective functions should be nonsingular, Pseudoinverse LDA (pLDA) was used to address the limitation, where the correct classification ratio using SBS+ pLDA achieved 95.5% for four emotional states of three subjects [117].

##### KNN

The authors of reference [104] used KNN (K = 4) to classify four emotions with the four features extracted from ECG, EMG, SC and RSP and yielded an average recognition rate of 82%. While in reference [118], by watching three sets of 10-min film clips eliciting fear, sadness, and neutral respectively, 14 features of 34 participants were extracted. Analyses used sequential backward selection and sequential forward selection to choose different feature sets for 5 classifiers (QDA, MLP, RBNF, KNN, and LDA). The models were assessed by four different cross-validation types. Among all these combinations, the KNN (K = 17) model achieved the best accuracy for both subject- and stimulus-dependent and subject- and stimulus-independent classification.

##### RF

In the work of [55], the authors used RF to classify five emotional states with features extracted from blood oxygen saturation (OXY), GSR and HR and yielded an overall correct classification rate of 74%.

### 4.3. Deep Learning Methods (Model-Free Methods)

#### CNN

**Figure 14:**
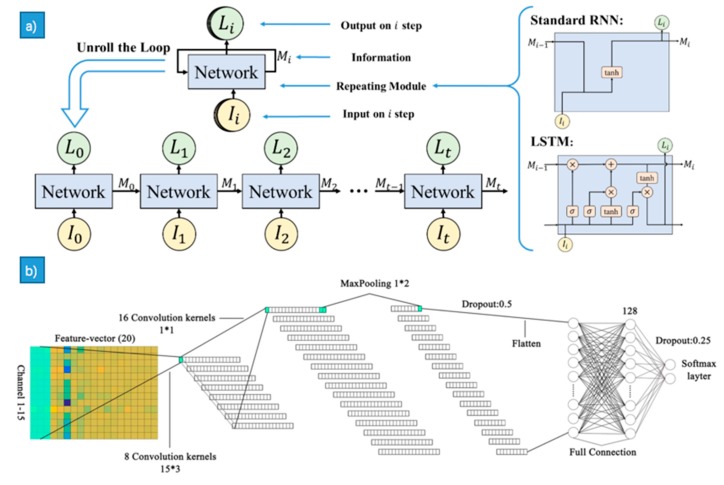
(**a**) The structure of standard RNN and LSTM; (**b**) The structure and settings of CNN.

Convolutional Neural Networks (CNNs), a class of deep, feed-forward artificial neural networks based on their shared-weights architecture and translation invariance characteristics, have achieved great success in image domain. They have been introduced to process physiological signals, such as EEG, EMG and ECG in recent years. Martinez et al. trained an efficient deep convolution neural network (CNN) to classify four cognitive states (relaxation, anxiety, excitement and fun) using skin conductance and blood volume pulse signals [119]. As mentioned in EEG, the authors of [23] used the Convolutional Neural Network (CNN) for feature abstraction (Figure 14b). In the work of [120], several statistical features were extracted and sent to the CNN and DNN, where the achieved accuracy of 85.83% surpassed those achieved in other papers using DEAP. In the work of [121], dynamical graph convolutional neural networks (DGCNN) was proposed, which could dynamically learn the intrinsic relationship between different EEG channels represented by an adjacency matrix, so as to facilitate more differentiated feature extraction and the recognition accuracy of 90.4% was achieved on the SEED database. 

#### DBN

The DBN is a complicated model which consists of a set of simpler RBM models. In this way, DBN can gradually extract the deep features of the input data. That is, DBN learns a deep input feature through pre-training. Wei-Long Zheng et al. introduced a recent advanced deep belief network (DBN) with differential entropy features to classify two emotional categories (positive and negative) from EEG data, where a hidden markov model (HMM) was integrated to accurately capture a more reliable emotional stage switching, and the average accuracies of DBN-HMM was 87.62%[122]. In the work of [123], DE features were extracted and DBN was applied in mapping the extracted feature to the higher-level characteristics space, where the highest accuracy of 94.92% for multi-classification was achieved. In the work of [124], instead of the manual feature extraction, the raw EEG, EMG, EOG and GSR signals were directly input to the DBN, where the high-level features according to the data distribution could be extracted. The recognition accuracies of 78.28% and 70.33% were achieved for valence and arousal on the DEAP database respectively.

#### PNN

PNN is a feed forward neural network based on the Bayesian strategy. PNN has the advantages of simple structure and fast learning ability, which leads to a more accurate classification with higher tolerance to the errors and noises. PNN was applied in the EEG emotion recognition with the sub band power features, but the correct classification rate of the PNN was slightly lower than that of the SVM (81.21% vs. 82.26% for valence; 81.76% vs. 82% for arousal). While the channels needed to achieve the best performance using PNN were much less than that of using SVM (9 vs. 14 for arousal; 9 vs. 19 for valence) [102].

#### LSTM

LSTM can deal with the vanishing gradient problem of RNN and can utilize the sequence of long-term dependencies and contextual information. In the reference [94], CNN was utilized to extract features from EEG and then LSTM was applied to train the classifier (Figure 14a), where the classifier performance was relevant to the output of LSTM in each time step. In the study by the authors of [27], RASM was extracted and then was sent to the LSTM to explore the timing correlation relation of signal, and an accuracy rate of 76.67% was achieved. In the work of [125], an end-to-end structure was proposed, in which the raw EEG signals in 5s-long segments were sent to the LSTM networks, in which autonomously learned features and an average accuracy of 85.65%, 85.45%, and 87.99% for arousal, valence, and liking were achieved, respectively. In the work of [126], the author proposed a model with two attention mechanisms based on multi-layer LSTM for the video and EEG signals, which combined temporal and band attentions. An average recognition accuracy of 73.1% and 74.5 for arousal and valence was achieved, respectively.

### 4.4. Model Assessment and Selection

#### 4.4.1. Evaluation Method

The generalization error of the classifier can be evaluated by experiments, where a testing set should be used to test the ability of the classifier to classify the new samples, and the testing error on the testing set could be viewed approximately as the generalization error.

##### Hold-Out Method

The dataset D is divided into two mutually exclusive sets. One is the training set S and the other is the testing set T. It’s necessary to maintain the consistency of the data distribution as much as possible. The experiments generally need to repeat several times with random division and then calculate the average value as the evaluation result. The 2/3~4/5 percent of the dataset are usually used for training and the remaining samples are used for testing.

##### Crossing-Validation Method

There are two kinds of crossing-validation methods which are usually used. One is k-fold crossing-validation. The other is leave-one-out. For k-fold cross-validation, the initial sampling is divided into K sub-samples. One sub-sample is used as the testing set, and the other K-1 samples are used for training. Cross-validation is repeated K times. Each sub-sample is validated once, and the average result of K times is used as the final result. 10-fold cross-validation is the most common method. Leave-one-out (LOO) uses one of the original samples as a testing set while the rest is left as training set. Although the results of LOO are more accurate, the time of training is too long when the dataset is large.

#### 4.4.2. Performance Evaluation Parameters

##### Accuracy

The accuracy rate and error rate are most commonly used in classification. The accurate rate means the proportion of samples that are correctly classified to the total samples. The error rate means the proportion of misclassified samples to the total samples.

##### Precision Rate and Recall Rate

The precision and recall rate can be defined using the following equations and Table 2.

(3) $$Precision-rate(P)=\frac{TP}{TP+FP}$$

(4) $$recall-rate(R)=\frac{TP}{TP+FN}$$

There is an opposite interdependency between recall and precision. If the recall rate of the output increases, its accuracy rate will reduce and vice versa.

##### F1

F1 is defined as the harmonic mean of the precision rate and the recall rate.

(5) $$F1=\frac{2*P*R}{P+R}$$

##### Receiver Operating Characteristic Curve (ROC)

The horizontal axis of ROC is false positive rate, the vertical axis of ROC is true positive rate.

(6) $$false-positive-rate(FPR)=\frac{FP}{TN+FP}$$

(7) $$true-positive-rate(TPR)=\frac{TP}{TP+FN}$$

**Figure 15:**
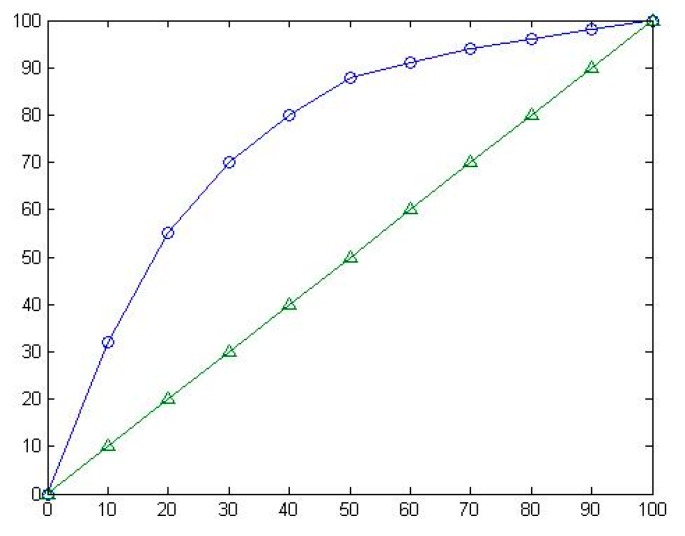
ROC.

It is easy to select the best threshold of the classifier with the ROC. The closer to the upper left corner the ROC is, the higher accuracy rate the test will be. It can be used to compare the classification ability of different classifiers. The ROC of the different classifiers can be plotted in the same coordinates to identify the pros and cons. The ROC which is closer to the upper left corner indicates that the classifier works better. It is also possible to compare the area under the ROC (AUC) and the classifier with the AUC. Likewise, the bigger AUC works better. The ROC is showed in Figure 15.

## 5. Database

### 5.1. Database for Emotion Analysis Using Physiological Signals (DEAP)

DEAP is a database for human emotion analysis [16]. It comprises the 32-channel EEG and 12 other peripheral physiological signals, including 4 EMG, 1 RSP, 1 GSR, 4 EOG, 1 Plethysmograph and 1T. The data was collected at a sample rate of 512 Hz, and some preprocessing processes were done. The signals were down sampled to128 Hz. EEG channels were applied a bandpass frequency filter from 4–45 Hz, and the EOG artifacts were removed from EEG. There were 32 participants in the database. Each of them watched 40 music videos with different emotional contents, each of which lasted one minute. Participants rated the videos with arousal, valence, dominance, liking after each trial. Arousal, valence and dominance were measured by the self-assessment manikins (SAM). The liking showed how much the video the subject liked with thumbs-up and thumbs-down.

**Figure 16:**
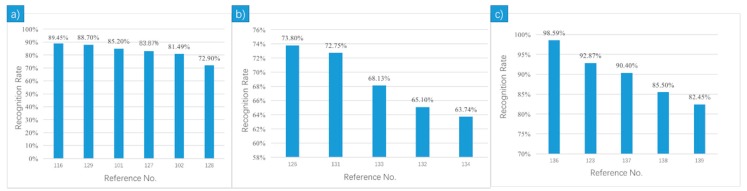
Comparative results on the same public accessible datasets: (**a**) DEAP; (**b**) MAHNOB; database; (**c**) SEED.

DEAP has been widely used in emotion recognition as it contains multiple physiological signals with reliable labels and exists for years. The mean recognition accuracies of the relevant studies on DEAP were plotted in Figure 16a. Different feature-extraction methods were adopted. Reference [116] achieved the highest mean accuracy 89.45% for three levels of valence and arousal due to the use of Gaussian process latent variable models (GP-LVM), through which latent points were extracted as dynamical features to train the SVM. In dynamic affective modeling, latent space features can describe emotional behaviors with less data. Bimodal Deep AutoEncoder network was used to generate high level features in [101], where the extracted features were then imported into a linear SVM and the highest accuracy rate of 85.2% was achieved. SVM was also used in [127]. Unlike [101], the statistical features such as mean, standard deviation, mean value of first order difference and so on were extracted from five bands of EEG signals to recognize four classes. The work of [128] divided the datasets into two parts: low familiarity and high familiarity. The best performance was achieved with unfamiliar songs, fractal dimension (FD) or power spectral density (PSD) features and SVM with a mean accuracy of 72.9%. Reference [129] segmented the last 40 s of each recording into 4 s, while [102] divided each 60 s trial into 14 segments with 8 s length and 50% overlapping. Reference [129] shows better accuracy using one-way ANOVA and semi-supervised deep learning approaches SDAE and DBN. The major classifier applied in the 6 references on DEAP was SVM.

### 5.2. MAHNOB Database

In order to facilitate the study of multimedia labels in new areas, a database of the responses of participants to the multimedia content was constructed [130]. 30 participants were recruited and were showed movies and pictures in the experiment. It comprises 32 channels EEG, RSP amplitude and skin T. The participants were asked to rate the stimulus on a scale of valence and arousal.

The mean recognition accuracies of the relevant studies on MAHNOB are plotted in Figure 16b. The model in reference [126] achieved the highest mean recognition accuracy of 73.8%, which was a novel multi-layer LSTM-RNN-based model with a band attention and a temporal attention mechanism to utilize EEG signals effectively and improve the model efficiency. Multi-modal fusion was applied in effect recognition by the authors of [131]. In decision-level classification fusion, regression-estimated weights fusion (W-REG) combined with recursive feature elimination (RFE) obtained the best results of 73% for valence and 72.5% for arousal. In [132], Neighborhood Components Analysis was applied to improve the KNN performance, achieving the highest classification accuracies of 66.1% for arousal and 64.1% for valence with ECG signal. In reference [133], 169 statistical features were inputted to the SVM using the RBF kernel, and a mean accuracy of 68.13% was obtained. The classification step was actualized on the Raspberry Pi III model B. Narrow-band (1–2 Hz) spectral power was applied, and ANOVA was used in feature selection followed by the SVM classifier, which reached an accuracy rate of 64.74% for arousal and 62.75% for valence, respectively [134].

### 5.3. SJTU Emotion EEG Dataset (SEED) 

The SEED database [135] contains EEG and eye movement of three different emotions (positive, neutral and negative). There were 15 participants (7 males, 8 females, mean: 23.27, STD: 2.37) in the experiment. 15 film clips, each of which lasted 4 min, were selected as stimuli. There were 15 trials for each experiment and totally 45 experiments in the dataset. The EEG signals was recorded from 62 channels at a sampling rate of 1000 Hz. Some preprocessing processes were done for the EEG data. The EEG data was down sampled at 200 Hz and was applied a bandpass frequency filter from 0–75 Hz. 

The classification results on SEED database are relatively better than others, as can be seen in Figure 16c. SEED was generally used in emotion recognition based on EEG. The Differential Entropy (DE) of five frequency bands are the most commonly used features in the SEED relevant references. In reference [136], the authors extracted features using Pearson Correlation Coefficient Matrix (PCCM) following the Differential Entropy (DE) and achieved the highest classification accuracy of 98.59% among these references. The authors of [137] proposes novel dynamical graph convolutional neural networks (DGCNN) and obtained the highest recognition accuracy of 90.4% for subject-dependent, as well as 79.95% for subject-independent experiments. Similarly, the authors of [123] obtained 5 s of DE in the beta frequency band, which was named emotional patches as feature sets, in which a DBN model stacked by three layers of RBM was proposed, and the highest accuracy rate of 92.87% was reached. In the work of [138], the authors found that with an accuracy rate of 82.56%, gamma band was more relative to emotional reaction compared with other single bands. A stacked autoencoder deep learning network was used for classification. The highest rate was achieved when DE in all frequency bands were inputted. A novel group sparse canonical correlation analysis (GSCCA) method was applied in [139] to select EEG channels and classify emotions. GSCCA model reached an accuracy rate of 82.45% with only 20 EEG channels, while the SVM needed to use 63 channels to get a similar result.

### 5.4. BioVid Emo DB 

The BioVid Emo DB [140] is a multimodal database. It comprises 3 physiological signals of SC, ECG, EMG. 86 participants were recruited and watched film clips to elicit five discrete basic emotions (amusement, sadness, anger, disgust and fear). They had to rate it for valence, arousal, amusement, sadness, anger, disgust and fear on nine points’ scales. In the study of [38], the authors extracted statistical features based on the wavelet transform from ECG signal and got a highest classification accuracy of 79.51%.

## 6. Summary

**Figure 17:**
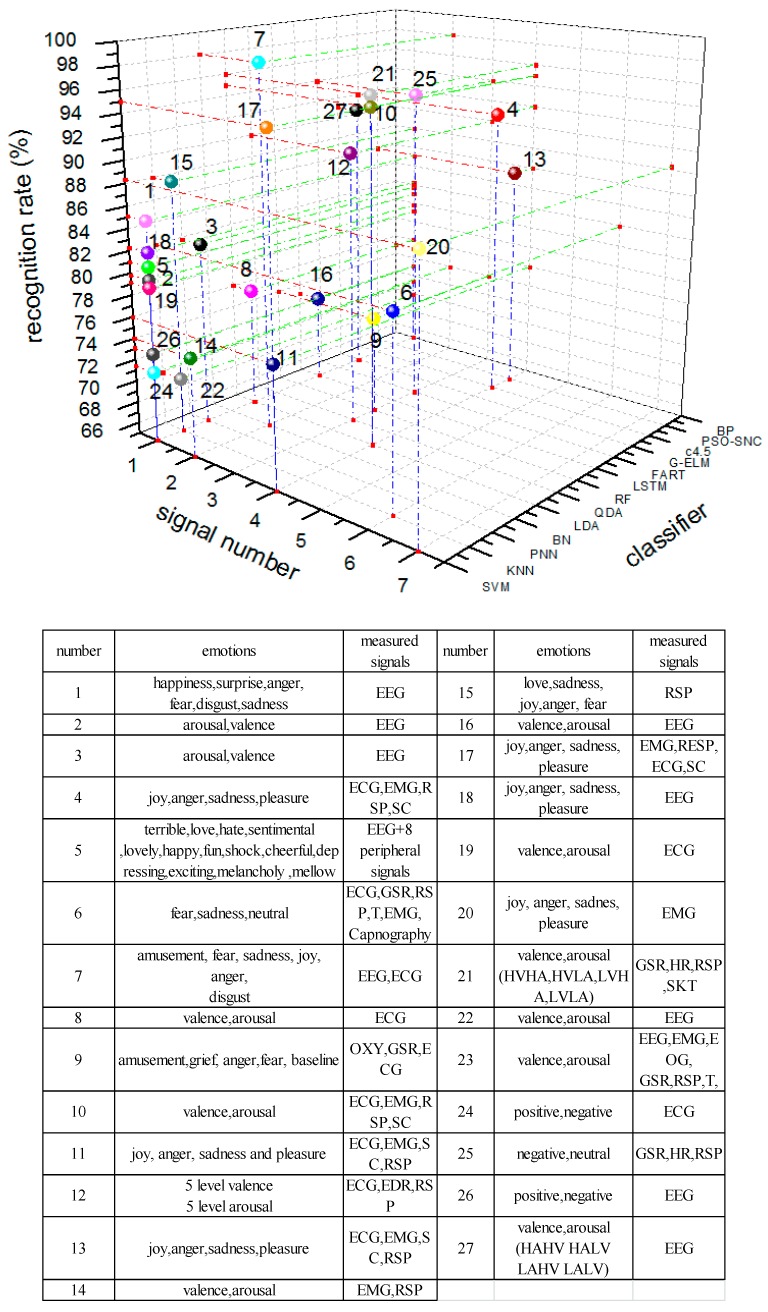
The comparation of recognition rate among previous research.

Table 3 summarized the previous studies in physiological signal-based emotion recognition, which lists the relevant information of: what kinds of stimuli were used, how many subjects were included, what emotions were recognized, what kinds of features and what kinds of classifier was chosen, as well as the recognition rate. Figure 17 shows the comparation among the previous research.

There are methods for emotions elicitation, such as images, games, music and films. As we can see from Table 3, the films are most commonly used, which require more cognitive participation and can induce the strong emotional feeling [143]. Compared to the films, the emerging VR scenes could be more vivid and immersive, thus might become a trend in emotional stimulation. The lengths of the stimuli response measures often last from 10 s to 120 s.

As for the trails and the involved participants, the number ranged from a few to dozens in the literatures, where the commonly used DEAP consisted of 32 participants and each contained 40 trials. Of course, more subjects and trials would help to improve the generalization ability of the model.

Since the physiological signals are non-stationary, the FFT is no longer applicable for physiological signals analysis, which targets for stationary signals. To deal with the problem, several feature extracting methods like STFT, DWT and EMD or robust features like HOC and FD have been proposed.

As for classifiers, the nonlinear classifiers (SVM with kernel, CNN, LSTM, Tree Models, BP, etc.) are more commonly used than the linear classifiers (LR, Perceptron, LDA, etc.). Compared to valence, identification rate of arousal is higher.

**Figure 18:**
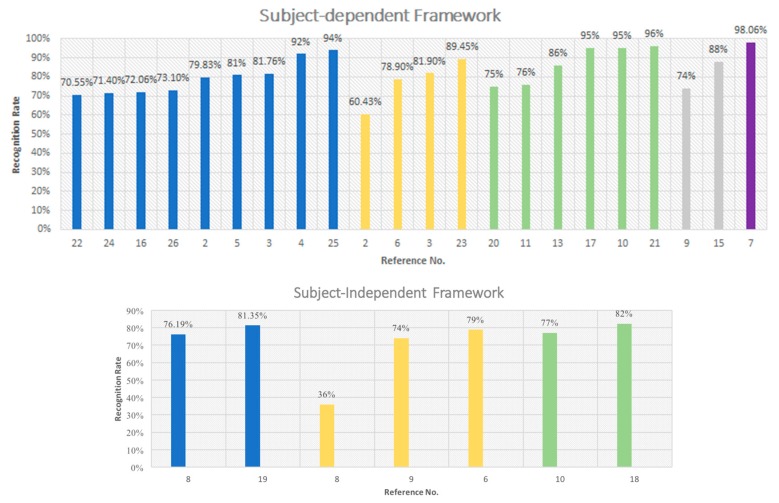
Subject-dependent and Subject-independent recognition rate. (The horizontal axis represents the same sequence number as the Table 3. Different colors represent different classification categories: Blue—two categories, yellow—three categories, green four—categories, grey—five categories, purple—six categories).

Figure 18 is an intuitive representation of Table 3 on the comparison of subject-dependent and subject-independent. Comparing subject-dependent and subject-independent settings, in subject-dependent training, N-fold cross-validation over each subject’s data is performed, and then the results over all the subjects are averaged. In subject-independent training, this processing was done by leave-one-subject-out cross-validation. The session of a particular subject was removed, and the algorithm was trained on the remaining trials of the other subjects and then applied to this subject’s data. It can be observed in the Figure 18 that it is hard to obtain high accuracy in the subject-dependent case. The leave-one-out setting is the conventional approach, but it is not very applicable to real-world applications, because it does not provide estimates of classification accuracy with unknown subjects. In order to find out the factors that affect recognition accuracy under different modality, we compare two aspects, respectively: (1) internal comparison and (2) comparison between two modes.

**(1) Internal Comparison**

The work of [37,102,103,141] all have ordinary performance respectively. This calls for improvement in applying an objective method for selecting a minimal or optimal feature subset, rather than ad hoc selected features. In the study of [141] (Ref No. 2 in Figure 18), the authors investigated feature vector generation from EEG signals, where only statistical features were considered through exhaustive test. Therefore, Feature vectors for the SVM classifier utilized a range of statistics-based measures, and the accuracy was about 79.83% for bipartition. A ReliefF-based channel selection algorithm was proposed [102] (Ref No. 3 in Figure 18) to reduce the number of used channels for convenience in practical usage. Similarly, power density was the one and the only feature that extracted from frequency domain. Although ReliefF was a widely used feature selection method in classification problems due to its effectiveness and simplicity of computation, the size of feature subset still limited the accuracy of classification. Ref [103] (No. 22 in Figure 18) and [37] (No. 24 in Figure 18) exhibited the recognition accuracy of 70.5% and 71.40% respectively.

Feature selection is a difficult optimization problem (NP-hard problem) for which there is no classical solving methods. In some cases, the feature vector comprised individual values, whereas in other cases the feature vector comprised a concatenation of values. So, some feature selection algorithms are adopted to propose new feature subsets, along with an evaluation measure which scores the different feature subsets. Some researchers used simple sequential feature selection algorithms (SFS and SBS) to improve the recognition rate. Another noteworthy data is from Ref [72] (No. 7 in Figure 18), where six emotions corresponding to joy, fear, sadness, pleasure, anger, and disgust were classified with an average accuracy of 98.06% using biological signals. The authors used a novel method of correcting the individual differences for each user when feeling the emotion, where the data associated with specific user were preprocessed to classify the emotion. Therefore, the weight of user-related specific channel was assigned to improve the classification accuracy.

**(2)  Comparison between two modes**

In contrast to many other studies, the authors of [117] (Ref No. 10 in Figure 18) and [118] (Ref No. 6 in Figure 18) paid special attention to evaluating performances in different test settings of both subject independence and subject dependence, in which they all exhibited satisfactory performances. To explore the reason lying behind this, we carried out a comparison to find the common practice. First of all, the same method in feature selection was used in these two papers (automated feature selection), which helped them achieve improved classification accuracy with a reduced feature subset. Sequential feature selection algorithms SFS and SBS were used. Secondly, they both chose LDA. It should also be noted that analyses in [118] showed only small improvements of the non-linear classifiers (QDA, MLP, RBNF, and KNN) over the linear classifier (LDA). Another noteworthy point was observed in [118]. There is relatively small decrease in classification accuracy from subject- and stimulus-dependent cross-validation over subject-independent cross-validation and stimulus-independent cross-validation to subject and stimulus-independent cross-validation. It demonstrated that three emotional states (fearful, sad, and neutral) can be successfully discriminated based on a remarkably small number of psychophysiological variables, by most classifiers, and independently of the stimulus material or of a particular person.

The authors of [29] (Ref No. 18 in Figure 18) demonstrated a high recognition rate with an averaged classification accuracy of 82.29%. Support vector machine was employed to classify four music-induced emotional states (joy, anger, sadness, and pleasure). In Table 3, studies using music-induced emotions generally exhibited higher accuracy than others. This might provide a different view point and new insights into music listening and emotion responses. We expect that further understanding the different stages of how the brain processes music information will make an impact on the realization of novel EEG-inspired multimedia applications. One the other hand, the problem of stimulus-dependent and stimulus-independent about the music pieces should be taken into account.

## 7. Problems and Future Work

This paper describes the whole framework of emotion recognition. A lot of efforts have been made in revealing the relationships between explicit physiological signals and implicit psychological feelings. However, there are still several challenges in emotion recognition based on physiological signals.

Firstly, obtaining high quality physiological data for affecting analysis requires a well-designed experimental setup. There are two common ways to elicit emotions: The most common setup is the standard lab setting in which subjects with earphones sit fairly motionless in front of a visible screen where the emotion stimuli materials are played. The lab environments are fixed, so that the data are noiseless and stable. On the other hand, the issue of obtaining genuine emotions which is dependent heavily on the emotion-stimulated materials is hard to deal with. So, more work should be done aiming at affective data generation. Special attentions need to be paid in how to choose some proper methods like VR equipment to induce direct and accurate targeted emotions that are close to real-world feeling. Another natural setup for gathering genuine emotions is in the real-world situations using the sensors of wearable devices. These devices are light-weight and hidden-recording with the capability of detecting the signals of skin conductivity, skin temperature, heart rate and some other emotion-related physiological parameters in a long period. But most studies focused on the short-time emotion recognition from seconds to minutes. It only classifies the instantaneous emotion state rather than a long-time emotion monitoring which may last for hours or days or even months. The main reason might be that it is difficult to accurately record the label of emotions of subjects continuously in the long period. It is so crucial for emotion classification in supervised learning because the labels of emotions are indispensable. There is still a long way to go in long-time emotional data collection and emotion recognition in the real world.

Secondly, as the stimulus materials are artificially selected, the labels of the materials are manually set, while human emotions vary from each other for the same thing, the ratings of the materials may have a large deviation. There are lots of factors effecting the emotions in the materials. In [144], the author showed that different induction modalities led to different physiological responses. There is no clear experimental paradigm that have been tested and verified to obtain high quality physiological data for affect analysis. It still requires much effort to form an experimental paradigm and build a huge open source database which should be included in the milestone for emotion recognition.

Thirdly, many researchers endeavor to find the most emotion-related features from physiological signals. There are time domain features like the statistical features, fractal dimension (FD), frequency domain features like PSD, PE and higher order spectra and time-frequency domain features extracted from the EMD and the WT. Though many features have been tried, there is still no clear evidence that what feature combinations of what physiological signal combinations are the most significantly relevant to emotion changes. Some of the work might depend on the research progress of human brain, especially the perspective of emotion generation mechanisms.

Fourthly, for most studies, the number of subjects is usually small, ranging from two or three up to thirty at most. Due to limited samples, the performance of the classifier with subjects who have not been trained would be poor. There are two approaches to solve this problem. One is to include more subjects from different ages and backgrounds. The other is to train the specific classifier for each user when there are few users because the classifier shows good performance when a subject has been trained.

Fifthly, emotion perception and experience lead to strong individual differences. The corresponding physiological signals thus alter to some extent. Current subject-independent recognition models are not yet advanced enough to be workable in realistic and real-time applications, which need further in-depth studies. Many studies relied on group analyses attempt to characterize common features across subjects using algorithms like ReliefF, SFS, SBS, TS, which usually regard variance among individuals as statistical noise.

The above approaches are to improve generalization by selecting robust features and filters, but the average identification accuracy is still far below user-dependent ones. More detailed investigation of such individual differences is still at a very early stage, although some are trying to minimize their effects. Individual differences are mainly reflected in the differences of signal baselines and emotional physiologies by different subjects. In order to make the emotional physiological data of different people comparable, the individual differences caused signal baseline variations should be normalized.

Sixth, the reliability of facial expressions cannot be guaranteed sometimes. However, when subjects are watching movie clips or stimulus fragments of people’s facial expressions, a phenomenon called ‘facial mimicry’ [145], which means a spontaneous and rapid facial EMG response in the same muscles involved in expressing the same positive or negative emotions induced by negative or positive emotional facial expressions, will appear. Research shows facial mimicry can facilitate empathy and emotional reciprocity [146]. According to this, even if subjects suppressed their facial expressions deliberately, facial EMG will still be useful. Physical responses to emotions such as facial mimicry and RSA (Respiratory Sinus Arrhythmia) can be modulated by different factors like childhood traumatic experiences [147,148], psychiatric diseases [149,150], and ingroup or outgroup membership [145]. The research on this aspect is helpful, because the best feature sets of emotion recognition can be selected for different groups according to the specific study purpose such as the psychological treatment of autistic patients and people who suffer from childhood trauma, which may require long-term and real-time monitoring of patient’s emotions. In order to improve the accuracy and reliability of existing emotion recognition methods, interdisciplinary research is necessary.

Seventh, there are several factors in the preprocessing and analytical procedures for choosing the classifiers. If the number of samples is small, only the linear classifiers are applicable. Additionally, it is necessary to split the data into smaller segments to obtain more training samples. Besides, it is reasonable to extract a relatively small number of features when the sample number is small. Regarding the emotion-classification framework, the preprocessing steps, as well as the analytical procedures between model-free vs. model-specific perspective, should be considered.

Finally, most emotion recognition systems are used to classify the specific emotion states, such as happiness, sadness. Studies on the evolution process of the emotion state are limited, which is not conducive to learning a person’s emotion changing process. We expect that the combinations of physiological signals, including EEG, ECG, GSR, RSP, EMG, HR, will lead to significant improvements in emotion recognition in the coming decade, and that this recognition will be critical to impart machines the intelligence to improve people’s life.

## Figures and Tables

**Table 1:** The relationship between emotions and physiological features *.

	Anger	Anxiety	Embarrassment	Fear	Amusement	Happiness	Joy
**Cardiovascular**							
HR	↑	↑	↑	↑	↑↓	↑	↑
HRV	↓	↓	↓	↓	↑	↓	↑
LF		↑		(--)		(--)	
LF/HF		↑			(--)		
PWA				↑			
PEP	↓		↓	↓	↑	↑	↑↓
SV	↑↓	(--)		↓		(--)	↓
CO	↑↓	↑	(--)	↑	↓	(--)	(--)
SBP	↑	↑	↑	↑	↑--	↑	↑
DBP	↑	↑	↑	↑	↑--	↑	(--)
MAP			↑	↑	↑--	↑	
TPR	↑			↓	↑	↑	(--)
FPA	↓	↓		↓	↓	↑↓	
FPTT	↓	↓		↓		↑	
EPTT		↓		↓		↑	
FT	↓	↓		↓	(--)	↑	
**Electrodermal**							
SCR	↑	↑		↑	↑		
nSRR	↑	↑		↑	↑	↑	↑
SCL	↑	↑	↑	↑	↑	↑--	(--)
**Respiratory**							
RR	↑	↑		↑	↑	↑	↑
Ti	↓	↓		↓--	↓	↓	
Te	↓	↓		↓		↓	
Pi	↑			↑		↓	
Ti/Ttot				↑	↓		
Vt	↑↓	↓		↑↓	↑↓	↑↓	
Vi/Ti						↑	
**Electroencephalography**							
PSD (α wave )	↑	↑		↓	↑	↑	↑
PSD (β wave)	↓				↑		
PSD (γ wave)				↓	↑	↑	↑
DE (average)	↑	(--)		↓		↑	↑
DASM (average)	(--)			↑	↓	↓	↓
RASM (average)	↑			↑		↓	

Note.* Arrows indicate increased (↑), decreased (↓), or no change in activation from baseline (−), or both increases and decreases in different studies (↑↓).

**Table 2:** The confusion matrix of classification.

True Situation	Prediction	
	Positive	Negative
Positive	true positive (TP)	false negative (FN)
Negative	false positive (FP)	true negative (TN)

**Table 3:** Summary of previous research.

No.	Author	Stimulus	Subjects	Subject Dependency	Emotions	Signals	Features	Classifiers	Recognition Rates
1	Petrantonakis P C, et al. [106]	IAPS	16 (9 males, 7 females)	No	happiness, surprise, anger, fear, disgust, sadness	EEG	FD, HOC	KNN, QDA, MD, SVM	85.17%
2	Samara A, et al. [141]	videos	32	Yes	arousal, valence	EEG	statistical features, PSD, HOC	SVM	Bipartition: 79.83% Tripartition: 60.43%
3	Jianhai Zhang et al. [102]	videos	32	Yes	arousal, valence	EEG	power	PNN, SVM	81.76% for PNN 82.00% for SVM
4	Ping Gong et al. [87]	music	-	Yes	joy, anger, sadness, pleasure	ECG, EMG, RSP, SC	statistical features Wavelet, EEMD, nonlinear	c4.5 decision tree	92%
5	Gyanendra Kumar Verma et al. [96]	videos	32	Yes	terrible, love, hate, sentimental, lovely, happy, fun, shock, cheerful, depressing, exciting, melancholy, mellow	EEG+8 peripheral signals	different Powers, STD and SE of detail and approximation coefficients.	SVM, MLP, KNN, MMC	EEG only:81% mixed with peripheral signals: 78%
6	Vitaliy Kolodyazhniy et al. [118]	film clips	34 (25 males, 19 females)	Both	fear, sadness, neutral	ECG, GSR, RSP, T, EMG, Capnography	HR, RSA, PEP, SBP, SCL, SRR, RR, Vt, pCO_2_, FT, ACT, SM, CS, ZM	KNN, MLP, QDA, LDA, RBNF	subject dependent:81.90% subject independent:78.9%
7	Dongmin Shin et al. [109]	videos	30	Yes	amusement, fear, sadness, joy, anger, and disgust	EEG, ECG	relative power, LF/HF	BN	98.06%
8	Foteini Agrafioti et al. [36]	IAPS and video game	44	No	valence, arousal	ECG	BEMD:Instantaneous Frequency, Local Oscillation	LDA	arousal: Bipartition76.19% C.36% valence: from 52% to 89%
9	Wanhui Wen et al. [55]	videos	-	No	amusement, grief, anger, fear, baseline	OXY, GSR, ECG	155 HR features and 43 GSR and first deviation GSR features	RF	74%,(leave-one-out) LOO
10	Jonghwa Kim et al. [117]	music	3	Both	valence, arousal	ECG, EMG, RSP, SC	110 features.	pLDA	subject dependent:95% subject independent:77%
11	Cong Zong et al. [100]	music	-	Yes	joy, anger, sadness and pleasure	ECG, EMG, SC, RSP	HHT:instantaneous frequency, weighted mean instantaneous frequency	SVM	76%
12	Gaetano Valenza et al. [35]	IAPS	35	No	5 level valence 5 level arousal	ECG, EDR, RSP	89 standard features, 36 nonlinear methods	QDA	>90%
13	Wee Ming Wong et al. [72]	music	-	Yes	joy, anger, sadness, pleasure	ECG, EMG, SC, RSP	32 features: mean, STD, breathing rateand amplitude, heartbeat, etc.	PSO of synergetic neural classifer (PSO-SNC)	SBS:86% SFS:86% ANOVA:81%
14	Leila Mirmohamadsadeghi et, al. [73]	videos	32	Yes	valence, arousal	EMG, RSP	slope of the phase difference of the RSA and the respiration	SVM	74% for valence, 74% for arousal and 76% for liking.
15	Chi-Keng Wu et al. [74]	flims clips	33	Yes	love, sadness, joy, anger, fear	RSP	EES	KNN5	88%
16	Xiang Li et al. [94]	videos	32	Yes	valence, arousal	EEG	CWT, CNN	LSTM	72.06% for valence, 74.12 for arousal
17	Zied Guendil et al. [95]	music	-	Yes	joy, anger, sadness, pleasure	EMG, RESP, ECG, SC	CWT	SVM	95%
18	Yuan-Pin Lin et al. [29]	music	26 (16 males,10 females)	No	joy, anger, sadness, pleasure	EEG	DASM, PSD, RASM	MLP, SVM	82.29%
19	Gaetano Valenza et al. [34]	IAPS	-	No	valence, arousal	ECG	spectral, HOS	SVM	79.15% for valence, 83.55% for arousal
20	Bo Cheng et al. [83]	-	-	Yes	joy, anger, sadnes, pleasure	EMG	DWT	BP	75%
21	Saikat Basu et al. [142]	IAPS	30	Yes	valence, arousal (HVHA, HVLA, LVHA, LVLA)	GSR, HR, RSP, SKT	mean, covariance matrix	LDA, QDA	98% for HVHA, 96% for HVLA, 93% for LVHA, 97% for LVLA
22	ingxin Liu et al. [103]	videos	32	Yes	valence, arousal	EEG	statistical features, PSD, HOC, Hjorth, FD, NSI, DWT, DA, DS, MSCE	KNN5, RF	69.9% for valence, 71.2% for arousal
23	Hernan F. Garcia et al. [116]	videos	32	Yes	valence, arousal	EEG, EMG, EOG, GSR, RSP, T, BVP	Gaussian process latent variable models	SVM	88.33% for 3 level valence, 90.56% for 3 level arousal
24	Han-Wen Guo et al. [37]	movie clips	25	Yes	positive, negative	ECG	Mean RRI, CVRR, SDRR, SDSD, LF, HF, LF/HF, Kurtosis, Kurtosis, entropy	SVM	71.40%
25	Mahdis Monajati et al. [56]	-	13 (6 males, 7 females)	Yes	negative, neutral	GSR, HR, RSP	GSR-dif = (GSR-max) − (GSR-base), mean-HR, mean-RR	Fuzzy Adaptive Resonance Theory	94%
26	Lan Z et al. [22]	IADS	5	Yes	positive, negative	EEG	FD, five statistical features, HOC, power	SVM	73.10%
27	Zheng W L et al. [25]	videos	47	Yes	valence, arousal (HAHV HALV LAHV LALV)	EEG	PSD, DE, DASM, DASR, DCAU	G extreme Learning Machine	69.67% in DEAP 91.07% in SEED
